# Application of Neural Network Algorithm Based on Principal Component Image Analysis in Band Expansion of College English Listening

**DOI:** 10.1155/2021/9732156

**Published:** 2021-11-12

**Authors:** Cailing Hao

**Affiliations:** School of Foreign Languages, Ningxia Normal University, Guyuan, Ningxia 756000, China

## Abstract

With the development of information technology, band expansion technology is gradually applied to college English listening teaching. This technology aims to recover broadband speech signals from narrowband speech signals with a limited frequency band. However, due to the limitations of current voice equipment and channel conditions, the existing voice band expansion technology often ignores the high-frequency and low-frequency correlation of the audio, resulting in excessive smoothing of the recovered high-frequency spectrum, too dull subjective hearing, and insufficient expression ability. In order to solve this problem, a neural network model PCA-NN (principal components analysis-neural network) based on principal component image analysis is proposed. Based on the nonlinear characteristics of the audio image signal, the model reduces the dimension of high-dimensional data and realizes the effective recovery of the high-frequency detailed spectrum of audio signal in phase space. The results show that the PCA-NN, i.e., neural network based on principal component analysis, is superior to other audio expansion algorithms in subjective and objective evaluation; in log spectrum distortion evaluation, PCA-NN algorithm obtains smaller LSD. Compared with EHBE, Le, and La, the average LSD decreased by 2.286 dB, 0.51 dB, and 0.15 dB, respectively. The above results show that in the image frequency band expansion of college English listening, the neural network algorithm based on principal component analysis (PCA-NN) can obtain better high-frequency reconstruction accuracy and effectively improve the audio quality.

## 1. Introduction 

English listening test is an important part of the English proficiency test and plays an important role in English proficiency learning. However, due to the limitations of the existing voice acquisition equipment, voice playback equipment, and communication conditions, there are some problems in the English listening audio, such as poor sound quality and natural damage, which have a great impact on the learning and evaluation of English listening [1]. Therefore, how to improve the sound quality of English listening through existing technical means has attracted more and more researchers' attention. At present, due to the limitations of channel bandwidth, coding mode, cost, and other factors in the communication network, English listening audio signals can only retain the low-frequency part (0.3 kHz–3.4 kHz) for transmission [2]. The lack of high-frequency signal directly leads to the reduction of timbre, naturalness, clarity, and intelligibility of the audio signal, which makes the sound lower and reduces the expression ability of original natural speech; it makes the listener unable to distinguish similar consonants, resulting in the decline of English listening recognition ability. Under the background that the existing communication system and communication network are difficult to be greatly improved and updated in a short time, the band expansion technology of recovering or expanding the high-frequency part lost due to factors such as channel bandwidth in the communication system has important practical significance for improving the overall sound quality of English listening audio and students' learning experience.

Audio signal band expansion technology means to artificially add certain frequency information to the reconstructed broadband audio at the receiving end by analyzing the signal characteristics of the original full band audio without affecting the network transmission and broadband signal source coding, so as to recover its high-frequency band components, to enhance the auditory quality and reproduce the ultrawide audio [3]. The traditional audio band expansion method is based on the statistical acoustic model. It uses the Gaussian mixture model (GMM) to describe the mapping relationship between low-frequency acoustic features and high-frequency acoustic features. In this way, there will be problems such as insufficient modeling accuracy and too smooth reconstruction of the high-frequency spectrum [4]. With the development of information technology, the neural network model with deep structure has attracted the interest of researchers. Compared with the traditional Gaussian mixture model, the neural network has better modeling ability for the nonlinear relationship between features. Although the frequency band method based on neural network can effectively predict and recover the high-frequency spectrum envelope and spectrum details, the existing models often use the methods of spectrum moving and spectrum folding to recover the detailed spectrum, ignore the correlation between high and low frequency of the audio, do not follow the evolution law of audio signal itself, and ignore the research on the nonlinear system generating the audio signal. This also leads to spectral distortion of the recovered audio signal. At the same time, the existing models are only suitable for low-dimensional data modeling and it is difficult to model the high-dimensional original spectrum envelope or amplitude spectrum features, which will lead to excessive smoothing of the recovered high-frequency spectrum, too dull subjective hearing, and insufficient expressiveness. In order to solve this problem, a neural network model (principal component analysis-neural network (PCA-NN)) based on principal component image analysis is proposed. Starting from the nonlinear characteristics of audio signal, the model reduces the dimension of high-dimensional data and realizes the effective recovery of the high-frequency detailed spectrum of audio signal in phase space.

In order to solve the problems of weak expressiveness, low recognizability, and spectrum distortion caused by the existing English listening audio band expansion technology, this paper studies and proposes a neural network algorithm based on principal component image analysis, which aims to effectively recover the high-frequency detailed spectrum of audio signal through the existing technical means and complete the expansion of English listening band, to improve the overall sound quality of English listening audio and students' learning experience. In Section 1, the paper briefly explains the research background and significance of English listening band expansion and introduces the overall framework and content arrangement of the article.  Section 2 briefly describes the research status of audio band broadening technology, discusses the problems to be solved in this field, and makes a general introduction to the research work and research methods of this paper.  Section 3 first introduces the model framework of PCA-NN based on principal component image analysis and then widens the English listening band based on the PCA-NN model. In Section 4, through the comparison of application experiments, the application feasibility of the PCA-NN model is studied.  Section 5 briefly summarizes the main conclusions of the article.

## 2. Related Work

In order to obtain bright and expressive audio services, many researchers have done a lot of work on how to make the broadband audio system obtain subjective hearing. As the most effective audio enhancement method, band expansion artificially adds high-frequency components to the reconstructed signal of the decoder without changing the source coding and network transmission, so as to realize the expansion of signal bandwidth [5]. Based on this idea, relevant scholars have proposed many band expansion solutions from two aspects of spectrum envelope and spectrum details. In 1994, Li et al. proposed the method of using statistical recovery function to predict the high-frequency spectrum, which preliminarily improved the quality of the reconstructed audio [6]. In the same year, with the help of the joint codebook of low-frequency feature and high-frequency feature spectral envelope to simulate the one-to-one mapping between them, Aboelmaged et al. proposed a spectral envelope estimation method based on codebook mapping [7], which reduced the distortion of the audio spectrum after expansion to a certain extent. On this basis, other scholars have proposed interpolation, soft decision, and split codebook mapping [8] to reduce the spectrum distortion caused by a single codebook. In 2000, Yao et al. proposed a spectral envelope estimation method based on the Gaussian mixture model [9]. GMM is used to approximate the joint probability density of high- and low-frequency features, and the estimation of high-frequency spectrum envelope is realized under the principle of minimum mean square error. In addition, Migenda et al. and Ge et al., respectively, use forward neural networks to estimate the high-frequency spectrum envelope [10, 11]. Weaving et al. compared the forward mapping network method with the codebook mapping method. The results showed that there was no significant difference in audio quality between the two methods, while the computational complexity of the forward neural network method decreased significantly [12]. These methods focus on exploring the correlation between high and low frequencies in the current audio frame, focusing on the display of the static characteristics of the spectrum. Gupta et al. used the hidden Markov model to simulate the time-domain dynamic evolution of the audio spectrum envelope [13] and introduced interframe correlation into spectrum envelope estimation. However, because this method only uses discrete states to simulate the time evolution of the actual audio spectrum, there is still dynamic distortion in the reconstructed audio.

With the rise in neural network technology, frequency band expansion technology based on neural network is also launched one after another. Fan et al. used shallow multilayer perceptron neural network, but its improvement effect is not obvious [14]. Peng et al. applied the shallow stochastic network to modeling of highly nonlinear mapping relationship between narrowband speech features and high-frequency speech feature parameters. This method can better preserve and reconstruct the details of the high-frequency spectrum [15]. Hassib et al. proposed simplified parameter bandwidth extension (SPBE) high-frequency reconstruction technology, which focuses on analyzing the correlation between high-frequency part and low-frequency score [16]. Lee et al. used the HBE method to stretch the low-frequency spectrum information to obtain the high-frequency spectrum details. However, there is a large gap between the reconstructed spectrum shape and the high-end components of the original audio [17]. Selecting the appropriate neural network to predict the signal generated by the nonlinear system can achieve high accuracy and can accurately restore the high-frequency spectrum characteristics.

To sum up, although many scholars have done a lot of work on audio band expansion and the existing methods can effectively predict and recover the high-frequency spectrum envelope and spectrum details, they ignore the correlation between high-frequency and low-frequency audio and the nonlinearity of audio signal, which makes the spectrum of recovered audio signal prone to distortion. At the same time, the existing models are only suitable for low-dimensional data modeling and it is difficult to model the high-dimensional original spectrum envelope or amplitude spectrum features, which will cause the recovered high-frequency spectrum to be excessively smooth, where the supervisor's listening sense is too dull and it is of insufficient expression. In view of this, PCA-NN, a neural network model based on principal component image analysis, is proposed. Starting from the nonlinear characteristics of the audio signal, the model reduces the dimension of high-dimensional data and realizes the effective recovery of the high-frequency detailed spectrum of audio signal in phase space.

## 3. Frequency Band Expansion of College English Listening Based on Principal Component Image Analysis-Neural Network (PCA-NN) Algorithm

PCA can be defined as the orthogonal projection of data on a low-dimensional linear space, which is called the principal subspace, so that the variance of the projected data is maximized, that is, the maximum variance theory. Equivalently, it can also be defined as a linear projection that minimizes the average projection cost, that is, the minimum error theory. Average projection cost refers to the average square distance between data points and their projections.

### 3.1. Neural Network Model Based on Principal Component Analysis (PCA-NN)

Principal component image analysis (PCA) is a method to reveal the internal relationship between multivariable indicators and large samples. It uses the idea of dimension reduction to simplify multiple indicators into a small number of comprehensive indicators, so as to reduce the dimension of processed samples and ensure the acquisition of the most important information [18]. Assuming that the original variable index is *a*_1_, *a*_2_,……, *a*_*m*_ and its principal component is *x*_1_, *x*_2_,……, *x*_*n*_(*n* < *m*), there are

(1) $$\begin{array}{c} \left\{\begin{array}{c} x_{1}=h_{11}a_{1}+h_{12}a_{2}+\cdots +h_{1m}a_{m},\\ \ldots \\ x_{n}=h_{n1}a_{1}+h_{n2}a_{2}+\cdots +h_{nm}a_{m}. \end{array}\right) \end{array}$$

**Figure 1:**
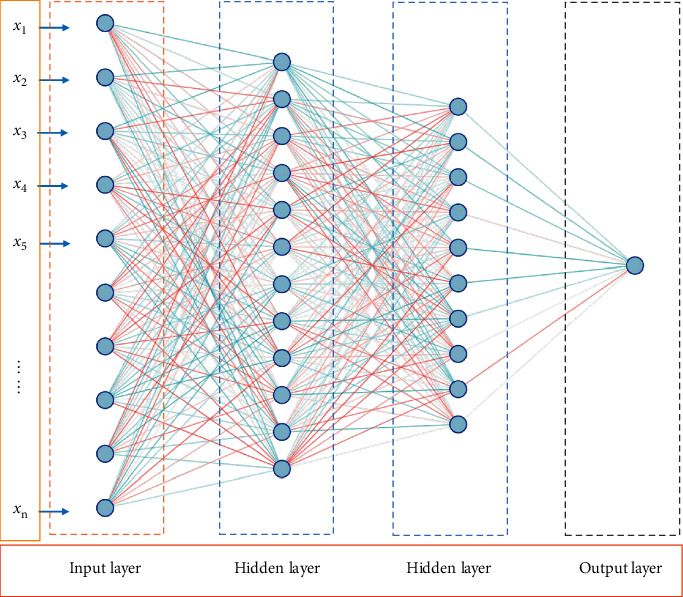
Neural network structure based on principal component image analysis.

In formula (1), *x*_1_, *x*_2_,……, *x*_*n*_ is called the first, second,…, and *n*th principal components of the original variable index *a*_1_, *a*_2_,……, *a*_*m*_ and *x*_1_, *x*_2_,……, *x*_*n*_ is linearly independent. The neural network structure based on principal component image analysis is shown in Figure 1.

As can be seen from Figure 1, after principal component image analysis, *n* principal components are selected to represent the original data information as the input of the neural network. The network structure includes three parts: input layer, hidden layer, and output layer. In the neural network, the input vector is

(2) $$\begin{array}{c} X^{k}={\left(x_{1}^{k},x_{2}^{k},\ldots ,x_{n}^{k}\right)}^{T}. \end{array}$$

In equation (2), *k*=1,2,……, *h*, where *h* represents the number of learning mode pairs, that is, number of groups of data present. The desired output vector for the corresponding input mode is

(3) $$\begin{array}{c} Y^{k}={\left(y_{1}^{k}\right)}^{T}. \end{array}$$

The net input vector of the middle hidden layer is expressed as

(4) $$\begin{array}{c} S^{k}={\left(s_{1}^{k},s_{2}^{k},\ldots ,s_{p}^{k}\right)}^{T}. \end{array}$$

The output vector is expressed as

(5) $$\begin{array}{c} B^{k}={\left(b_{1}^{k},b_{2}^{k},\ldots ,b_{p}^{k}\right)}^{T}. \end{array}$$

In equation (5), P is the number of cells in the hidden layer, the net input vector of the output layer is expressed as *L*^*k*^=(*l*_1_^*k*^)^*T*^, and the actual output vector is expressed as *C*^*k*^=(*c*_1_^*k*^)^*T*^. The connection weight from the input layer to the hidden layer is expressed as

(6) $$\begin{array}{c} W=\left\{v_{ij}\right\},\;\left(i=1,2,\ldots ,n,j=1,2,\ldots ,p\right). \end{array}$$

The connection weight from the hidden layer to the output layer is expressed as

(7) $$\begin{array}{c} V=\left\{v_{j1}\right\},\;\left(j=1,2,\ldots ,p\right). \end{array}$$

**Figure 2:**
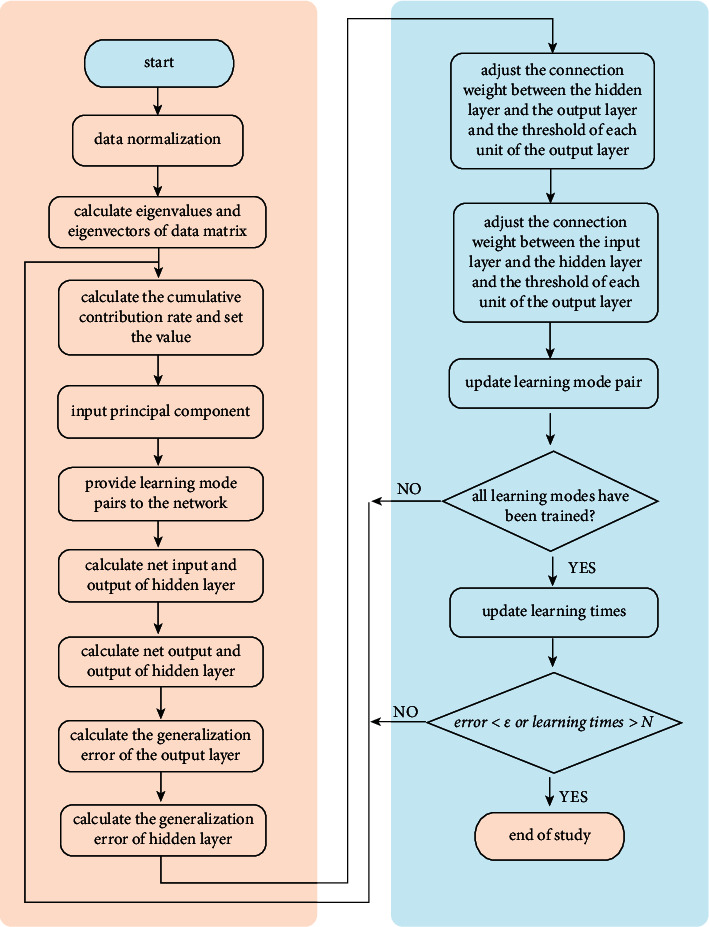
Neural network algorithm flow based on principal component image analysis.

The flow description of neural network algorithm based on principal component image analysis is shown in Figure 2.

As can be seen from Figure 2, the main flow of neural network algorithm based on principal component image analysis first requires normalizing the original data [19], converting all the original data into values between [0–1], and calculating the correlation coefficient of the matrix:

(8) $$\begin{array}{c} R=\left[\begin{array}{cccc} r_{11} & r_{12} & \ldots & r_{1p}\\ r_{21} & r_{22} & \ldots & r_{2p}\\ \vdots & \vdots & \vdots & \vdots \\ r_{p1} & r_{p2} & \ldots & r_{pp} \end{array}\right]. \end{array}$$

In formula (8), *r* is the correlation coefficient between the original variable and and its calculation formula is

(9) $$\begin{array}{c} r_{ij}=\frac{\sum_{k=1}^{n}\left(a_{ki}-\bar{a_{i}}\right)\left(a_{kj}-\bar{a_{j}}\right)}{\sqrt{\sum_{k=1}^{n}{\left(a_{ki}-\bar{a_{i}}\right)}^{2}\sum_{k=1}^{n}{\left(a_{kj}-\bar{a_{j}}\right)}^{2}}}. \end{array}$$

Then, the eigenvectors and eigenvalues are calculated, and the eigenequation |*λI* − *R*|=0 is solved. The eigenvalues are obtained by the Jacobian method and arranged according to their size order, namely, *λ*_1_ ≥ *λ*_2_ ≥ ⋯≥*λ*_*p*_ ≥ 0. The eigenvectors *e*_*i*_ (*i*=1,2,…, *p*) corresponding to the eigenvalue *λ*_*i*_ are obtained, respectively, requiring ‖*e*_*i*_‖=1. The principal component image contribution rate and cumulative contribution rate are calculated. The calculation formula of principal component contribution rate is

(10) $$\begin{array}{c} \frac{\lambda_{i}}{\sum_{k=1}^{p}\lambda_{k}},\;\left(i=1,2,\ldots ,p\right). \end{array}$$

The calculation formula of cumulative contribution rate is

(11) $$\begin{array}{c} \frac{\sum_{k=1}^{i}\lambda_{k}}{\sum_{k=1}^{p}\lambda_{k}},\;\left(i=1,2,\ldots ,p\right). \end{array}$$

Through repeated experiments, the optimal cumulative contribution rate is determined. Finally, *n* principal components are obtained, *n* principal components are input into the neural network, and a learning mode pair (*X*^*k*^, *Y*^*k*^) is randomly selected to provide to the network. The net output and input of neurons in the hidden layer are calculated, and the calculation formulas are as follows:

(12) $$\begin{array}{c} s_{j}=\sum_{i=1}^{n}w_{ij}x_{i}^{k}-\theta_{j}\;j=1,2,\ldots ,p,\\ b_{j}^{k}=f\left(s_{j}^{k}\right)\;j=1,2,\ldots ,p. \end{array}$$

The actual output and net input of each neuron in the output layer are calculated, and the calculation formulas are as follows:

(13) $$\begin{array}{c} l_{1}^{k}=\sum_{j=1}^{p}v_{j1}b_{j}^{k}-\gamma_{1},\\ c_{1}^{k}=f\left(l_{1}^{k}\right). \end{array}$$

According to the given expected output, the correction error of each neuron in the output layer is calculated. The calculation formula is

(14) $$\begin{array}{c} d_{1}^{k}=\left(y_{1}^{k}-c_{1}^{k}\right)f^{\prime }\left(l_{1}^{k}\right). \end{array}$$

A learning mode pair is randomly selected to provide to the network until all learning mode pairs have completed training. It is judge whether the network global error *E* meets the accuracy requirements, that is, *E* < *ε*. If it meets the algorithm requirements, it will end; if not, it will continue.

(15) $$\begin{array}{c} E=\frac{1}{d}\sum_{o=1}^{d}{\left(C_{o}-Y_{o}\right)}^{2}. \end{array}$$

In equation (15), *d* is the training time, *C*_*d*_ is the training output of the *o*th training, and *Y*_*d*_ is the expected output value of the *o*th training. Finally, the network learning time is updated. If it is less than the specified time, the cycle is continued; if the specified number of times is met, it ends.

### 3.2. Band Expansion of College English Listening Based on the PCA-NN Model

**Figure 3:**
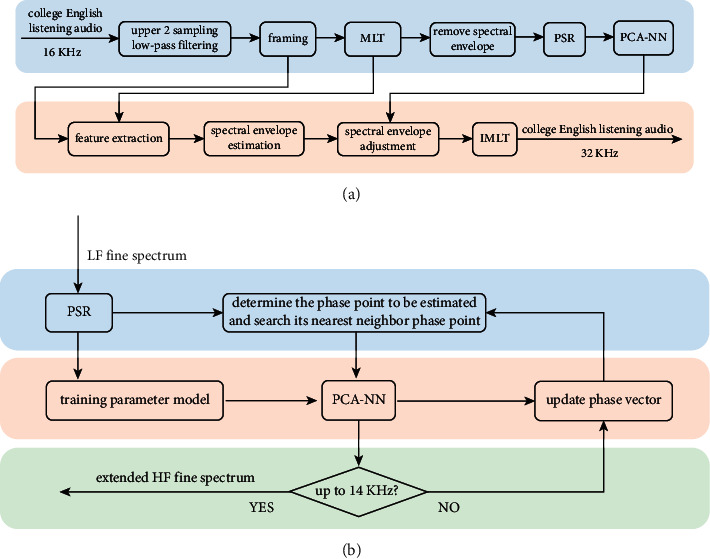
Band expansion of college English listening based on the PCA-NN model. (a) Principle block diagram of audio frequency band expansion of college English listening based on PCA-NN. (b) Principle block diagram of fine spectrum prediction based on PCA-NN.

The detailed spectral characteristics of audio directly determine the timbre characteristics of the audio. The traditional audio band expansion technology ignores the correlation between high and low frequencies of audio and the nonlinearity of audio signal system, which leads to spectrum distortion and poor auditory effect of the restored audio signal [20]. To solve these problems, starting from the nonlinearity of audio signal, this chapter uses the PCA-NN model to effectively recover the high-frequency detailed spectrum of audio signal in phase space. Its basic principle is shown in Figure 3.

Figure 3 shows the basic principle of band expansion of college English listening based on the PCA-NN model. First, we input the unexpanded audio with a sampling rate of 16 kHz and a bandwidth of 7 kHz. After the upper two sampling processes and low-pass filtering, the signal is divided into frames according to the Hamming window overlapped by 50% of the frame length of 20 ms, and the MLT (modulated lapped transformation) of the modulated overlapped Hamming window is performed to obtain the MLT parameter *C*_*mlt*_(*i*),  *i*=0 ~ 279, below 7 kHz. These parameters are divided into 7 subbands according to 40 frequency points in each subband, then the MLT parameters are subband enveloped to obtain the low-frequency detailed spectrum parameter *x*(*k*),  *k*=0 ~ 280, of the current frame, and then the principal component image analysis method is used to extract the detailed spectrum parameters for the purpose of dimensionality reduction, Then, the phase space parameters of the one-dimensional audio frequency domain sequence of each frame are calculated by using the phase space reconstruction algorithm, and the phase space reconstruction of broadband frequency sequence is realized [21].

After realizing the phase space reconstruction of the audio frequency domain sequence, the PCA-NN is used to fit and predict the nonlinear system. In Figure 3(b), the space is preliminarily fitted based on the low-frequency phase points and the detailed spectrum parameters closest to the low-frequency and the high-frequency domain sequence are predicted. In the prediction process, the new phase points constructed from the predicted frequency domain sequence are continuously introduced into the training of the original network, which can effectively predict the subsequent high-frequency domain sequence while enriching the network training samples. The specific prediction steps are as follows:

1. 280 MLT parameters are obtained from each frame of the broadband audio signal by MLT, and 280 − (*m* − 1)*τ* phase points are obtained by phase space reconstruction.
2. The embedding dimension *m* of the phase space is used to determine the number of neurons in the input layer of the network, and the number of neurons in the output layer is set to 1, which is used to predict the high-frequency series one by one.
3. The phase point *X*(*n*)={*x*(*n*), *x*(*n*+*τ*), *x*(*n*+2*τ*),…, *x*(*n*+(*m* − 1)*τ*)} obtained by phase space reconstruction is used as the input layer signal after dimensionality reduction by principal component image extraction. The next MLT value *x*(*n*+(*m* − 1)*τ*+1) of the highest dimensional component of each phase point is used as the desired output layer signal. The PCA-NN training algorithm is used to train the network. Thus, the network weights *w* and *V* are obtained.
4. The last phase point *X*(*N* − (*m* − 1)*τ* − 1) of low-frequency MLT sequence is used as the input, and the output value of PCA-NN used in the 281st MLT value estimation, so as to reconstruct a new phase point *X*(*N* − (*m* − 1)*τ*). The new phase point and the original low-frequency phase point are introduced into the training of neural network at the same time, and this step is repeated until all high-frequency MLT coefficients are predicted, so as to expand the high-frequency fine spectrum.

## 4. Research on the Application Effect of Neural Network Algorithm Based on Principal Component Image Analysis in College English Listening Band Expansion

### 4.1. Band Expansion Test of College English Listening Based on the PCA-NN Model

**Figure 4:**
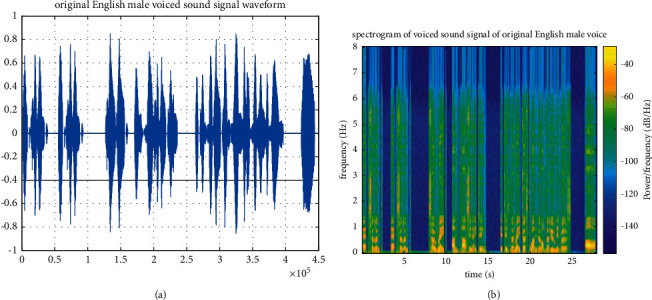
English male-voiced original sound signal. (a) Original sound signal waveform of the English male voiced sound signal. (b) Spectrogram of English male voiced original sound signal.

In order to test the expansion ability of the PCA-NN model for college English listening audio, this paper selects a section of English male-voiced audio and English male standard audio as audio evaluation signals for the expansion test [22]. Firstly, the voiced sampling frequency of English male voice is 32 kHz, and its original sound signal waveform and spectrogram are shown in Figures 4(a) and 4(b), respectively.

**Figure 5:**
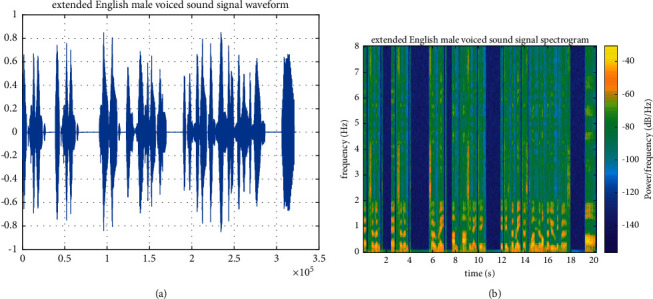
English male-voiced sound signal based on the PCA-NN model. (a) Sound signal waveform of English male-voiced sound signal after expansion. (b) Spectrogram of English male-voiced sound signal after expansion.

After expanding the audio frequency band through the PCA-NN model, the waveform and spectrogram of extended audio are obtained, as shown in Figures 5(a) and 5(b), respectively.

Figure 5 shows the voiced audio signal of boys in English after expansion using the PCA-NN model. It can be seen that the details of the high-frequency spectrum are clearer and richer after expansion and the change trend of high-frequency spectrum energy is maintained while effectively recovering the details of the high-frequency spectrum.

**Figure 6:**
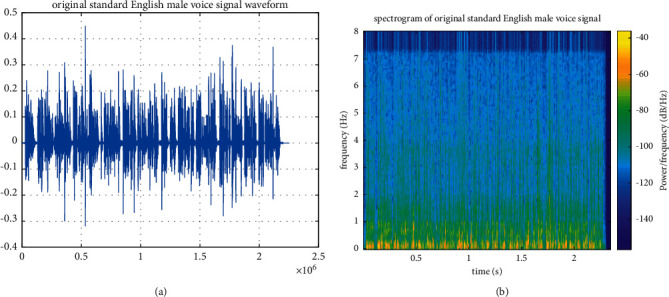
Standard English male-voiced signal. (a) Original sound signal waveform of the standard English male voice. (b) Spectrogram of the standard English male-voiced sound signal.

In order to test the adaptability of PCA-NN model, a section of English male-voiced standard audio is selected as the audio evaluation signal for the expansion test. The audio sampling frequency is 48 kHz. The waveform and spectrum of the original sound signal are shown in Figures 6(a) and 6(b), respectively.

**Figure 7:**
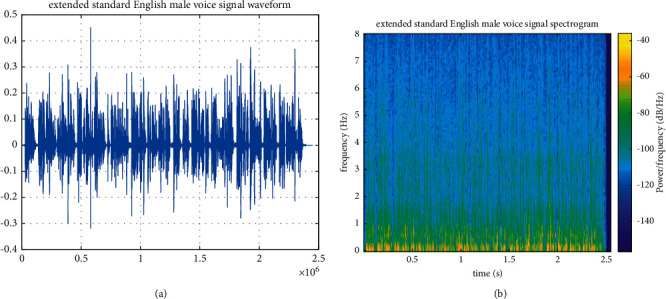
Standard English male-voiced signal based on PCA-NN. (a) Extended waveform of the standard English male-voiced signal. (b) Extended spectrogram of the standard English male-voiced signal.

After expanding the audio frequency band through the PCA-NN model, the waveform and spectrogram of the extended audio are obtained, as shown in Figures 7(a) and 7(b), respectively.

Figure 7 shows the standard audio signal of boys in English obtained after expansion using the PCA-NN model. It can be seen that the details of high-frequency spectrum are clearer and richer after expansion and the change trend of high-frequency spectrum energy is maintained while effectively recovering the details of the high-frequency spectrum.

The results of these two groups of test experiments show that the PCA-NN model proposed in this paper has good adaptability for both voiced audio and standard audio English listening. The results can meet the expected requirements of expanding the frequency band, and the audio quality after expansion is improved to a certain extent.

### 4.2. Performance Comparison between the PCA-NN Model and Mainstream Audio Band Expansion Model

In this section, the PCA-NN detailed spectrum nonlinear prediction algorithm proposed in this paper will be compared with the existing mainstream frequency band expansion algorithm from the subjective, objective, and log spectral distortion (LSD) perspectives to evaluate that the audio signals are excerpted from college English listening audio.

**Figure 8:**
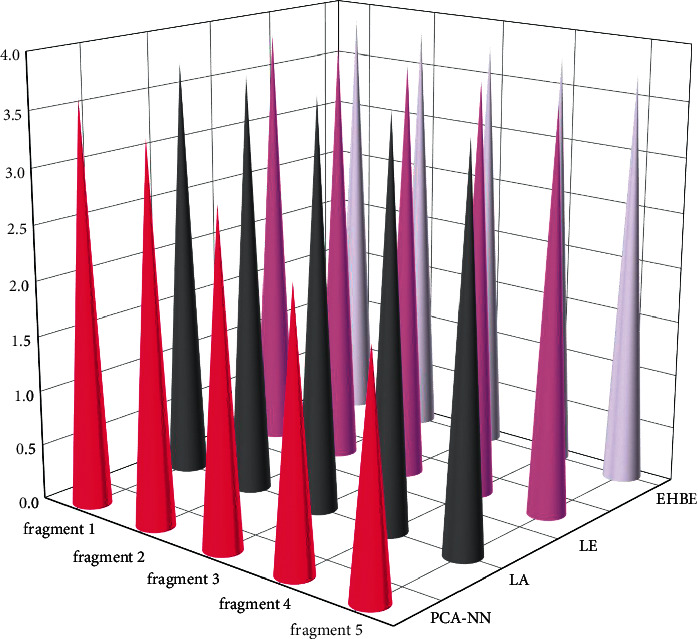
Objective evaluation ODG scores of PCA-NN and mainstream audio expansion algorithms.

The objective evaluation standard adopts the PEAQ test tool designed based on ITU-R bs.1387-1 standard. The test is an audio quality perception evaluation algorithm proposed by the International Telecommunication Union. Its main evaluation parameter is objective difference grade (ODG). The value range of ODG is 0∼4. The lower the score, the better the audio effect; and every 0.1 decrease in the ODG score indicates that the audio quality has been significantly improved. In the evaluation process, this paper compares the performance with the local adaptive nonlinear prediction method La (local adaptability), wired extrapolation Le (linear extrapolation), and effective high-frequency band extension algorithm EHBE (efficient high-frequency band extension) [23–25]. We select 5 different English listening materials as the audio evaluation object and obtain the ODG score of the extended audio through objective evaluation, as shown in Figure 8.

From the objective evaluation in Figure 8, it can be seen that the nondetailed spectrum scoring result of PCA-NN algorithm based on principal component image analysis proposed in this paper is better than that of other mainstream algorithms. PCA-NN can better improve the audio quality when expanding the audio frequency band of English listening.

**Figure 9:**
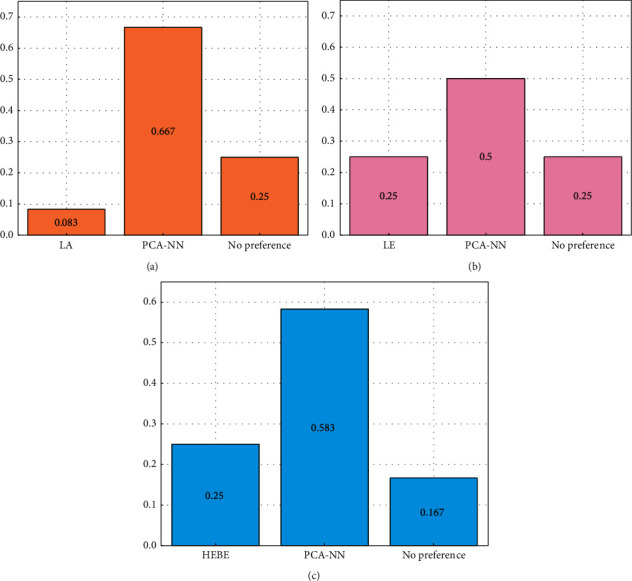
Subjective preference auditory test of PCA-NN and other mainstream audio extension algorithms. (a) La and PCA-NN. (b) Le and PCA-NN. (c) EHBE and PCA-NN.

In the supervisor test, the subjective preference auditory test method (A/B test) is adopted. Similarly, five different English listening materials are selected as the evaluation audio object. The tester is required to choose the preferred one from the UWB audio obtained by the two extension algorithms, and there is little difference between the two. In this paper, 9 people are selected for the supervisor test and the test results are shown in Figure 9.

Figure 9 abscissa represents PCA-NN and other mainstream audio expansion algorithms, and ordinate represents the test results of each algorithm. From the subjective evaluation in Figure 9, it can be seen that the audio after band expansion based on PCA-NN has obvious sound quality improvement and was preferred by a large number of people compared with the extended audio of other algorithms.

**Figure 10:**
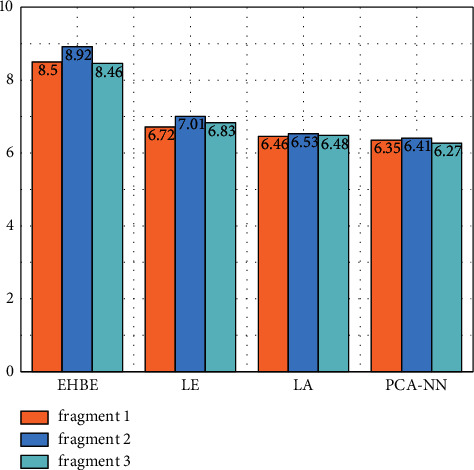
Log spectrum distortion test of PCA-NN and other mainstream algorithms.

In the log spectrum distortion test, the selected test data shall be aligned with the original super-bandwidth audio in the time domain, resampled to 32 kHz, and the whole LSD data shall be taken as its objective quality measure [26]. The test results are shown in Figure 10.

Figure 10 abscissa represents PCA-NN and other mainstream algorithms, and ordinate represents the log spectrum distortion test result score of each algorithm. From the log spectrum distortion test results in Figure 10, it can be seen that PCA-NN algorithm can obtain smaller LSD for three different college English listening audios. Therefore, PCA-NN can obtain better high-frequency reconstruction accuracy. Compared with EHBE, Le, and Le, the average LSD is reduced by 2.286 dB, 0.51 dB, and 0.15 dB respectively.

## 5. Conclusion

The existing audio expansion technology ignores the correlation between high and low frequencies of speech, resulting in excessive smoothing of the recovered high-frequency spectrum, too dull subjective hearing, insufficient expressiveness, and so on. In this paper, a neural network model based on principal component image analysis (PCA-NN) is proposed. Starting from the nonlinear characteristics of the audio signal, the model reduces the dimension of high-dimensional data and realizes the effective recovery of high-frequency detailed spectrum of the audio signal in phase space. The results show that PCA-NN based on principal component image analysis is better than other audio expansion algorithms in subjective and objective evaluation. In the log spectrum distortion evaluation, PCA-NN algorithm obtains smaller LSD. Compared with EHBE, Le and La, the average LSD is reduced by 2.286 dB, 0.51 dB, and 0.15 dB, respectively. Therefore, in the band expansion of college English listening audio, the algorithm PCA-NN based on principal component image analysis can obtain better high-frequency reconstruction accuracy, effectively improve the audio quality, and enhance the listener's auditory feeling.

## Data Availability

The data used to support the findings of this study are available from the corresponding author upon request.

## References

[1] Yang X. (2018). Analysis of college entrance examination English listening audio materials based on Adobe audit. *Overseas English*.

[2] Guo L., Wang S., Yin J., Wang Y., Yang J., Gui G. (2021). Federated user activity analysis via network traffic and deep neural network in mobile wireless networks. *Physical Communication*.

[3] Ramesh V. P., Baskaran P., Krishnamoorthy A., Damodaran D., Sadasivam P. (2019). Back propagation neural network based big data analytics for a stock market challenge. *Communications in Statistics-Theory and Methods*.

[4] Sun J., Tian Z., Fu Y., Geng J., Liu G. (2021). Digital twins in human understanding: a deep learning-based method to recognize personality traits. *International Journal of Computer Integrated Manufacturing*.

[5] Ait Hammou B., Ait Lahcen A., Mouline S. (2020). Towards a real-time processing framework based on improved distributed recurrent neural network variants with fast text for social big data analytics. *Information Processing & Management*.

[6] Li H. (2019). Spectrum analysis method based on fractional lower order statistics. *Journal of Tianjin Vocational and Technical Normal University*.

[7] Aboelmaged M., Mouakket S. (2020). Influencing models and determinants in big data analytics research: a bibliometric analysis. *Information Processing & Management*.

[8] Peng L., Peng M., Liao B., Huang G., Li W., Xie D. (2018). The advances and challenges of deep learning application in biological big data processing. *Current Bioinformatics*.

[9] Yao Y., Wang J., Long P., Xie M., Wang J. (2020). Small‐batch‐size convolutional neural network based fault diagnosis system for nuclear energy production safety with big‐data environment. *International Journal of Energy Research*.

[10] Migenda N., Möller R., Schenck W. (2021). Adaptive dimensionality reduction for neural network-based online principal component analysis. *PLoS One*.

[11] Ge Y., Ding Z., Meng W. (2020). Uniaxial negative thermal expansion and band renormalization in monolayer Td−MoTe2 at low temperature. *Physical Review B*.

[12] Weaving D., Beggs C., Dalton-Barron N., Jones B., Abt G. (2019). Visualizing the complexity of the athlete-monitoring cycle through principal-component analysis. *International Journal of Sports Physiology and Performance*.

[13] Gupta D., Shekhawat H. S. (2021). High‐band feature extraction for artificial bandwidth extension using deep neural network and H ∞ optimisation. *IET Signal Processing*.

[14] Fan J., Chow T. W. (2019). Exactly robust kernel principal component analysis. *IEEE transactions on neural networks and learning systems*.

[15] Peng H., Wang H., Du B. (2020). Spatial temporal incidence dynamic graph neural networks for traffic flow forecasting. *Information Sciences*.

[16] Hassib E. M., El-Desouky A. I., Labib L. M., El-Kenawy E.-S. M. (2020). WOA + BRNN: an imbalanced big data classification framework using Whale optimization and deep neural network. *Soft Computing*.

[17] Lee W. J., Mendis G. P., Triebe M. J., Sutherland J. W. (2020). Monitoring of a machining process using kernel principal component analysis and kernel density estimation. *Journal of Intelligent Manufacturing*.

[18] Mondal S., Nandi G., Pal P. K. (2021). Parametric optimization of TIG welding of duplex stainless steel without filler rod by PCA method. *IOP Conference Series: Materials Science and Engineering*.

[19] Konishi T. (2020). Principal component analysis of coronaviruses reveals their diversity and seasonal and pandemic potential. *PLoS One*.

[20] Cui Z., Li F., Zhang W. (2019). Bat algorithm with principal component analysis. *International Journal of Machine Learning and Cybernetics*.

[21] Gao P. P., Li Y. P., Huang G. H., Su Y. Y. (2021). An integrated Bayesian least-squares-support-vector-machine factorial-analysis (B-LSVM-FA) method for inferring inflow from the Amu Darya to the Aral Sea under ensemble prediction. *Journal of Hydrology*.

[22] Nushi M., Orouji F. (2020). Investigating EFL teachers’ views on listening difficulties among their learners: The case of Iranian context. *SAGE Open*.

[23] McCormack S., Jones B., Scantlebury S., Collins N., Owen C., Till K. (2021). Using principal component analysis to compare the physical qualities between academy and international youth rugby league players. *International Journal of Sports Physiology and Performance*.

[24] Jedamski R., Epp J. (2021). Non-destructive micromagnetic determination of hardness and case hardening depth using linear regression analysis and artificial neural networks. *Metals*.

[25] Schreiber J. B. (2021). Issues and recommendations for exploratory factor analysis and principal component analysis. *Research in Social and Administrative Pharmacy*.

[26] Meng Z., Chen W. (2020). Automatic music transcription based on convolutional neural network, constant Q transform and MFCC. *Journal of Physics: Conference Series. IOP Publishing*.

